# Preparation of magnetic mesoporous silica from rice husk for aflatoxin B1 removal: Optimum process and adsorption mechanism

**DOI:** 10.1371/journal.pone.0238837

**Published:** 2020-09-10

**Authors:** Yanan Li, Ren Wang, Zhengxing Chen, Xiuping Zhao, Xiaohu Luo, Li Wang, Yongfu Li, Fei Teng

**Affiliations:** 1 Key Laboratory of Carbohydrate Chemistry and Biotechnology, Ministry of Education, Jiangnan University, Wuxi, China; 2 National Engineering Laboratory for Cereal Fermentation Technology, Jiangnan University, Wuxi, China; 3 Jiangsu Provincial Research Center for Bioactive Product Processing Technology, Jiangnan University, Wuxi, China; 4 School of Food Science and Technology, Jiangnan University, Wuxi, China; Qatar University, QATAR

## Abstract

The liquid foodstuffs such as edible oil products remain a problem of excessive aflatoxin B1 (AFB1) content. This paper focused on the preparation of magnetic mesoporous silica (MMS) from rice husk ash for the removal of AFB1 in oil system. The MMS preparation process, adsorption conditions, structural characteristics, and adsorption mechanism were investigated. The optimum conditions for MMS preparation were pH 11.0 and 80°C for 24 h. The characterization results showed that magnetic particles were successfully embedded in the MMS and had high responsiveness to a magnetic field, which was advantageous for recyclability. The MMS had ordered uniform channels with a specific surface area of 730.98 m^2^/g and pore diameter of 2.43 nm. The optimum adsorption conditions were 2 h at 20°C. For AFB1 with an initial concentration of 0.2 μg/mL, the MMS adsorption capacity was 171.98 μg/g and the adsorption rate was 94.59%. The MMS adsorption isotherm fitted the Langmuir model well under the assumption of monolayer AFB1 adsorption with uniformly distributed adsorption sites on the MMS surface. The maximum amount of AFB1 adsorbed according to the Langmuir isotherm was 1118.69 μg/g. A quasi-second-order kinetic model gave a better fit to the process of AFB1 adsorption on MMS. The values of Δ*H* (−19.17 kJ/mol) and Δ*G* (−34.09, −34.61, and −35.15 kJ/mol at 283, 293, and 303 K, respectively) were negative, indicating that AFB1 adsorption on MMS was a spontaneous exothermic process. The results indicated that MMS was a promising material for AFB1 removal in oil system, and this study will serve as a guide for practical MMS applications.

## Introduction

Aflatoxins (AFs) were a group of toxic metabolites with similar chemical structures that were produced by fungi. They have been found in a wide range of crops such as maize, peanut, walnut, and their derived products [1]. The International Agency for Research on Cancer (IARC) classified AFs as class 1 human carcinogens [2, 3]. Twelve AFs types have been identified: AFB1, AFB2, AFG1, AFG2, AFM1, AFM2, AFP1, AFQ, AFH1, AFGM, AFB2a, and aflatoxicol. Aflatoxin B1 (AFB1) was listed as one of the strongest carcinogens by the IARC because of its high toxic, teratogenic, carcinogenic, and mutagenic effects [4]. According to an analysis of grains and forage in China, the AFs contamination rate was as high as 90% in bulk grain varieties [5]. AFB1 could not be destroyed by the temperatures used in typical processing treatments. One study found that 9.9% (184/1854) of AFB1-contaminated foodstuffs had AFB1 concentrations above the limitation of detection and that home-made peanut oil had the highest AFB1 concentration, with a mean value of 38.74 ± 47.45 μg/kg [6]. AFB1 contaminations of liquid foodstuffs such as edible oil products remain a problem and may have fatal effects on human health. Therefore, methods for safe and effective removal of AFB1 from liquid foodstuffs have received widespread attention.

Methods for AFB1 removal include chemical, biological, and physical approaches. Among these, the addition of adsorbents to reduce the bioaccessibility of AFB1 in feeds [7, 8] and foods was regarded as an effective and economical method. Sodium coumarin salt or ammonium salt, which easily dissolved in water, could be formed by the reaction of alkali and AFB1 in chemical alkali detoxification, which was suitable for AFB1 removal in oil systems. However, this had gradually been replaced with a more complicated process that required large equipment investment and was associated with high cost. It was difficult to remove a chemical reagent that was water-soluble because the reagent formed a residue that affected food quality [9, 10]. Biological methods were suitable for fermentation systems, but their application in liquid food systems was limited [11–13]. Physical adsorption was a common method for removing AFB1 from food [14, 15], but silicate adsorbents were not reusable because they could not be separated from the material effectively, so the applications in liquid foodstuffs were limited. Therefore, it was of great significance to identify adsorbents that were easy to separate from food materials and that could effectively remove AFB1 from liquid foodstuffs.

Mesoporous SiO_2_ (MS) had a small particle size. It was easy to suspend in the liquid phase, and thus it was difficult to effectively separate in liquid systems. Magnetic MS (MMS) had the characteristics of MS and magnetic particles and it could be rapidly and effectively separated by the application of an external magnetic field [16]. Some researchers have synthesized MMS materials with different structures, including embedded, core-shell, and hollow core-shell structures. MMS could be synthesized via an in situ method and a two-step method. In the two-step method, MS and magnetic particles were combined in a certain way to obtain MMS. Using tetraethyl orthosilicate (TEOS) as the silica source, MMS was prepared via an in situ method using iron nitrate [17] or Fe_3_O_4_ particles [18, 19]. MMS was a composite material with both a large surface area and abundant MS active sites with the advantage of easy separation of magnetic material [20, 21], and thus had high application value for removal of mycotoxins from liquid foodstuffs. As TEOS was expensive, searches for alternative cheap silica sources were ongoing.

Rice husk ash was a byproduct of the burning of rice husk for energy generation. The ash yield from rice husk was approximately 20% and the main ash component was SiO_2_. SiO_2_ in rice husk ash was amorphous according to X-ray diffraction (XRD) analysis [22]. Amorphous SiO_2_ had good activity, so rice husk ash was an ideal silica source. Si-OH groups on the surface had high reactivity and could be used to introduce active groups into the pore surface or skeleton for functionalization. At present, the main application of rice husk ash was as amorphous silica, which was used in the preparation of cement, concrete, carbon white, silica gel, water glass, and molecular sieves. Most studies using rice husk ash as a silica source have prepared zeolites rather than MS. High-silica ZMS-5 zeolites with a specific surface area of 397 m^2^/g have been synthesized using tetrapropylammonium bromide as a template and rice husk ash as the silica source [23]. The MMS materials MCM-41, MCM-48, and SBA-15 were synthesized using rice husk ash as a silica source, and surface Si-OH groups were functionalized and modified to obtain MMS with good CO_2_ adsorption capacity [24]. MS material (R-MCM) made from rice husk ash had a surface area of 1347 m^2^/g, a pore volume of 0.906 cm^3^/g, and a maximum removal efficiency for methyl blue of 99% [25]. The rate of methyl blue adsorption on magnetic material (MRHA) made from Fe_3_O_4_ and functionalized rice-husk ash was high, with a high *q*_m_ (150.5 mg/g) [26]. Sodium silicate solution extracted from rice husk has been used to synthesize various MS materials with a specific pore structure (mesocellular forms and hexagonal nanochannel structures), pore size (3–60 nm), surface area (297–895 m^2^/g), and pore volume (0.81–1.77 cm^3^/g) [27]. There was a wealth of rice husk ash raw materials in China, which could be used to prepare MMS as an ideal silicon source. This application would improve the utilization of rice husk ash, reduce environmental pollution, and effectively improve food safety by removing AFB1 from liquid foodstuffs.

In this study, we investigated a process for preparing MMS using rice husk ash as a silica source for AFB1 adsorption in a liquid phase system. The adsorption conditions were optimized and the adsorption mechanism was analyzed. The resulting MMS had good adsorption properties in a liquid phase system and could effectively remove AFB1 from liquid foodstuffs and enhance food safety. This application could also improve the utilization of rice husk ash and reduce environmental pollution. Furthermore, optimization of the AFB1 adsorption process and analysis of the adsorption mechanism might serve as a guide for practical MMS applications.

## Materials and methods

### Materials

Rice husk was obtained from Cofco Engineering Technology Company (Wuxi, China) and stored at room temperature before treatment. Cooking oil was purchased at supermarkets. Ultrapure water (resistivity ≥18 MΩ/cm) was generated using a Millipore-Q SP system (Millipore, Bedford, MA, USA) and prefiltered through 0.22-μm filters. The AFB1 reference standard (2,3,6a,9a-tetrahydro-4-methoxycyclopenta[c]furo[2,3:4,5]furo[2,3-h]chromene-1,11-dione; C_17_H_12_O_6_; ≥98% purity) was purchased from Alexis Corporation (Lausen, Switzerland). HPLC-grade methanol, toluene, and acetonitrile were purchased from J&K Scientific (Zhejiang, China). All other analytical grade chemicals and reagents were obtained from Sinopharm Chemical Reagent Company (Beijing, China).

### Synthesis of MMS nanoparticles

The procedure for the synthesis of MMS involved three steps. The first was the preparation of sodium silicate solution [28]. A certain amount of rice husk was washed three times and then dried at 50°C for 24 h. After grinding, the powders were boiled for 2 h in a solution of HCl (2 mol/L). The HCl treatment effectively removed metal ions remaining in the rice husk and dissolved most of the hemicellulose and a small amount of cellulose, and thus increased the silica content [27]. The precipitated product was washed with distilled water and then dried 12 h at 50°C. All dried samples were calcined for 6 h at 550°C. The resulting white powder and NaOH (4:5, w/w) were carefully weighed and dissolved in 250 mL of distilled water at 80°C to form sodium silicate solution.

The second step was the synthesis of Fe_3_O_4_ nanoparticles. We used the co-precipitation method described by Hong [29], with some modifications. Deionized water (150 mL) and N_2_H_4_·H_2_O (2 mL) were put into a three-necked 250-mL round-bottom flask equipped with a mechanical stirrer and agitated for 30 min to eliminate oxygen. Aqueous solutions of FeCl_3_ and FeSO_4_ were transferred into the flask at a Fe^3+^/Fe^2+^ ratio of 1.75:1, and then 8 mL of aqueous ammonia (25%, v/v) was quickly syringed into the flask with vigorous stirring. The solution was then maintained at 80°C for 30 min. The precipitate was filtered and washed with deionized water and anhydrous ethanol for 10 times, dried under vacuum for 24 h, and then ground.

The third step was the synthesis of MMS nanoparticles. Magnetic Fe_3_O_4_ nanoparticles (200 mg) were ultrasonicated for 30 min in a solution of hexadecyltrimethyl ammonium bromide (CTAB) (100 mL, 0.02 mol/L). The suspension was heated to 80°C under the mechanical stirring and then the sodium silicate solution (50 mL) obtained from step 1 was slowly added. After further stirring for 30 min, 1 mol/L HCl solution was added dropwise to the mixture to adjust the pH to 11. After an additional stirring for 2 h, the mixture was kept in a water bath for 24 h at 80°C. The precipitated product was washed with distilled water three times and then dried at 50°C. Finally, the CTAB surfactant was eliminated via calcination for 2 h at 550°C. The reddish powder obtained was considered to be MMS nanoparticles [18]. MS nanoparticles without Fe_3_O_4_ were prepared in a similar manner. CTAB acted as a template in the preparation of mesoporous materials [30]. It formed a hexagonal stacked liquid-crystal phase via self-assembly when its concentration exceeded the critical micelle concentration. The silica material was hydrolyzed and polymerized around the liquid-crystal phase to form an inorganic-organic compound. The CTAB was then removed via calcination and the silica was retained, with the spaces previously occupied by CTAB molecules forming a mesoporous structure [25].

### Determination of AFB1

We determined AFB1 concentrations according to GB 5009.22–2016 (National Food Safety Standard for the Determination of Aflatoxin B and G in Food) with some modifications. AFB1 solution (1 mL, 0.2 μg/mL) was blow-dried with nitrogen at 30°C and added to 200 μL of *n*-hexane and 100 μL of trifluoroacetic acid as the derivatization reagent. The sample was rapidly agitated for 15 s, derivatized for 30 min at 40°C, and dried under a stream of nitrogen at 30°C. The water-acetonitrile solution (1 mL, 85:15, v/v) was added to dissolve the residue, and the sample was centrifuged at 8,000 rpm for 5 min after agitation for 15 s. The supernatant was filtered through a 0.22 μm membrane and transferred to a chromatographic vial for analysis. AFB1 concentrations were determined using an Agilent 1260 series HPLC system (Agilent Technologies, Palo Alto, CA, USA) equipped with an autoinjector, a quaternary solvent delivery system, and a fluorescence detector. The excitation and emission wavelengths were set at 360 and 440 nm, respectively. Chromatographic separation was achieved on an Agilent ZORBAX SB-C18 column (Agilent Technologies). The mobile phase consisted of methanol-water (35:65, v/v). The injection volume was 10 μL and the flow rate was 1 mL/min.

### Characterization

MMS (0.1 g) prepared under optimum conditions was dispersed in 200 mL of distilled water. After 1 min, a magnet was applied to determine the separation performance. Fourier-transform infrared spectroscopy was carried out to identify specific functional groups in the adsorbent. Samples were pressed into KBr pellets and then analyzed with a Nicolet IS10 spectrometer over the wavenumber ranged from 400 to 4000 cm^−1^. XRD was performed on a Bruker AXS Advance instrument using copper as the target with K radiation at 40 kV and 40 mA. The 2θ range was 1–80° at a goniometer speed of 0.02%. To measure nitrogen adsorption-desorption isotherms, samples were degassed at 200°C for 2.5 h before experiments on a JW-BK6 analyzer. The BET (particle surface adsorption) and BJH (pore size distribution) methods were used to calculate the specific surface area, pore diameter, and pore size distribution of the samples [28].

### Adsorption process

MMS prepared under optimum conditions was used for single-factor tests of AFB1 adsorption in oil. The effect of the adsorption time and temperature on the unit adsorption capacity and adsorption rate of AFB1 in oil was investigated to determine the best conditions.

Cooking oil (10 mL) containing 0.2 μg/mL AFB1 was placed in a sample bottle and 11 mg of MMS was added. The bottle was placed on an air shaking platform (HYG-A, Taicang Experimental Equipment Factory, Suzhou, China) and shaken at 150 rpm for 5 h at 20°C. After centrifugation at 4,000 rpm for 10 min, the supernatant was removed and its AFB1 content was determined via HPLC. The unit adsorption capacity and AFB1 adsorption rate were calculated as follows:

$$q_{e}=\frac{(C_{0}-C_{e})\times V}{m}$$ (1)

$$AFB1\;\mathrm{adsorption}\;\mathrm{rate}=\frac{C_{0}-C_{e}}{C_{0}}\times 100$$ (2)

where *q*_*e*_ is the amount of AFB1 adsorbed per unit weight of adsorbent (μg/g), *C*_*o*_ is the initial concentration of AFB1 (μg/mL), *C*_*e*_ is the concentration of AFB1 in the supernatant at equilibrium (μg/mL), *V* is the solution volume (mL), and *m* is the mass of adsorbent (g).

### Adsorption isotherm

AFB1 adsorption isotherms were investigated in the batch equilibrium experiments. A known weight of the adsorbents was placed into a vial and 10 mL of AFB1 solution at different initial concentrations (0.05, 0.1, 0.15, 0.2, 0.4, 0.6, 0.8, and 1.0 μg/mL) was added respectively. The vials were shaken for 24 h at temperatures of 283, 293, and 303 K. The AFB1 concentration after reaching equilibrium was determined by HPLC. Langmuir and Freundlich isotherm models were used to describe the equilibrium adsorption according to the following relationships [31, 32]:

$$\frac{C_{e}}{q_{e}}=\frac{1}{q_{\max}}C_{e}+\frac{1}{K_{L}q_{\max}}$$ (3)

$$\ln q_{e}=\ln K_{F}+\frac{1}{n}\ln C_{e}$$ (4)

where *q*_*e*_ is the amount of AFB1 adsorbed per unit weight of adsorbent at equilibrium (μg/g), *C*_*e*_ is the concentration of AFB1 in the supernatant at equilibrium (μg/mL), *K*_*L*_ is the Langmuir constant (L/mg), and *q*_*max*_ is the maximum AFB1 capacity (μg/g). *K*_*F*_ and n are the Freundlich constants referring to adsorption capacity and adsorption intensity, respectively. Nonlinear regression analysis was performed using Origin Pro 9.0 to estimate the values of *q*_*max*_, *K*_*L*_, *n*, and *K*_*F*_.

### Adsorption thermodynamic parameters

Thermodynamic parameters could reveal whether adsorption was a physical or chemical process and whether it was exothermic or endothermic. The MMS-AFB1 adsorption mechanism was analyzed and the apparent thermodynamic functions were calculated via the adsorption equilibrium constant method [33]. Table 1 shows the relationship between the isothermal model parameters and *K*_*a*_; the parameters calculated were the apparent thermodynamic parameters. The changes in adsorption enthalpy (Δ*H*), Gibbs free energy (Δ*G*), and adsorption entropy (Δ*S*) could be calculated according to the van’t Hoff equation, Gibbs formula, and Gibbs-Helmholtz equation [34, 35] using formulas (5)–(7):

$$\ln C_{e}=-K_{0}+\frac{\Delta H}{RT}$$ (5)

$$\Delta G=-RT\ln k_{\alpha }$$ (6)

$$\Delta S=\frac{\Delta H-\Delta G}{T}$$ (7)

where *K*_*0*_ is a constant, *T* is the absolute temperature (K), and *R* is the ideal gas constant [8.314 J/(mol·K)].

**Table 1:** Relationship between isothermal model parameters and equilibrium constant.

Model type	Parameter	Relationship between isthomal model parameters and K_a_
Langmuir	*q*_*max*_、*K*_*L*_	*K*_*L*_ = *K*_*a*_
Freundlich	*K*_*F*_、n	*K*_*F*_^*n*^ = *K*_*a*_

### Adsorption kinetics

The AFB1 adsorption on MMS in oil was investigated at 20°C. The medium was cooking oil containing 0.2 μg/mL AFB1. The unit adsorption capacity and AFB1 adsorption rate were determined after adsorption times of 0.5, 1.0, 1.5, 2.0, 2.5, and 3.5 h. According to the results, quasi-first-order and quasi-second-order kinetic models were used for fitting to obtain the relationship between the reaction time and the sample adsorption capacity, and to analyze possible adsorption behavior. The specific model formulae are as follows [26]:

$$q_{t}=q_{e}(1-e^{-k_{1}t})$$ (8)

$$q_{t}=\frac{q_{e}^{2}k_{2}t}{1+k_{2}q_{e}t}$$ (9)

where *q*_*t*_ is the amount of AFB1 adsorbed after reaction time *t* (mg/g), *k*_*1*_ is the rate constant for the quasi-first-order kinetic reaction (min^−1^), and *k*_*2*_ is the rate constant for the quasi-second-order kinetic reaction (g/(mg·min)).

### Data analysis

Experiments were repeated three times. Excel 2013 and Origin Pro 9.0 were used for drawing, and SPSS 17.0 was used for ANOVA (mean values with different letters were significantly different at *p* < 0.05). Results were presented as the mean value plus or minus the standard deviation.

## Results and discussion

In preliminary experiments, the unit adsorption capacity of MMS for AFB1 was significantly higher in oil (90 μg/g) than in water (20 μg/g), indicating the strong adsorption of AFB1 by MMS in oil and weak adsorption in water. This was mainly because the MMS surface had a large number of Si-OH groups with a strong polarity, while AFB1 had a weak polarity. According to the similarity compatibility principle, MMS was more likely to combine with water molecules in highly polar water systems and with AFB1 in nonpolar oil systems. Therefore, the adsorption of AFB1 in this study was investigated in oil systems.

### Optimization of the MMS preparation process

The effective specific surface area, pore diameter, and surface charge distribution of the adsorbent affected the adsorption of AFB1 [36]. The reaction time, temperature, and pH during MMS preparation had important effects on the specific surface area, pore diameter, and channel structure [37]. To obtain MMS with good adsorption properties, we selected the reaction time, temperature, and pH for MMS preparation for optimization.

#### Effect of reaction time

The effects of the reaction time on the MMS structure and AFB1 adsorption capacity were studied at 25°C and pH 11.0. Table 2 shows that the specific surface area, unit adsorption capacity, and AFB1 adsorption rate of MMS increased with the reaction time up to 24 h, as the reaction time increased to 24 h, the specific surface area increased by nearly 100%, and the unit adsorption capacity and AFB1 adsorption rate increased by 70%. There were no significant changes in the three parameters for reaction times > 24 h. The results could be attributed to the transition of the mesoscopic structure of ordered MS from a lamellar phase to a six-part phase [38] and a gradual increase in the specific surface area. Thermal treatment for 24 h could enhance the hydrothermal stability of mesoporous materials, leading to an increase in adsorption stability and preventing structural collapse [37]. Therefore, we identified 24 h as the optimum reaction time for MMS preparation.

**Table 2:** Effect of reaction time on the specific surface area and adsorption to AFB1 of MMS.

Reaction time (h)	Adsorption capacity (μg/g)	Adsorption rate (%)	Specific surface area (m^2^/g)
0	89.96±4.04^a^	49.48±2.22^a^	307.15±10.11^a^
4	123.65±4.65^b^	68.01±2.56^b^	471.74±10.45^b^
8	143.56±2.76^c^	78.96±1.52^c^	603.50±20.63^c^
12	140.15±4.80^c^	77.08±2.64^c^	614.26±12.29^c^
18	145.29±2.33^c^	79.91±1.28^c^	622.44±8.77^c^
24	159.92±3.14^d^	87.96±1.72^d^	642.88±9.91^d^
30	155.57±2.29^d^	85.57±1.26^d^	644.32±8.27^d^

Results were presented as mean ± standard deviation. Mean values with different letters were significantly different at p < 0.05.

#### Effect of pH

As shown in Table 3, pH had significant effects on the MMS structure and AFB1 adsorption. It affected the polymerization of the silicate, which had an impact on the MS channel structure [37]. At neutral pH, the specific surface area was at a minimum because the speed of silicate polymerization was higher than that of hydrolysis. Therefore, the precipitate was separated quickly, leading to irregular pore structures. At lower or higher pH, there was sufficient time for MS to form a regular structure because of the slower speed of silicate polymerization [39]. In addition, at higher pH, the silica source dissociated into ionic-state SiO_2_ oligomers, contributing to enhanced interaction between the silica source and the surfactant. Thus, the pore structure was more regular and the specific surface area was greater. However, at pH 12.0, the silica source had a highly negative charge, which impedes pore wall thickening [38]. Therefore, the MMS specific surface area was smaller and the adsorption and hydrothermal stability were poorer. At pH 11.0, the unit adsorption capacity and AFB1 adsorption rate were at their maximum (161.87 μg/g and 89.03%, respectively). Therefore, pH 11.0 was chosen as the optimum preparation condition.

**Table 3:** Effect of pH on the specific surface area and adsorption to AFB1 of MMS.

pH	Adsorption capacity (μg/g)	Adsorption rate (%)	Specific surface area (m^2^/g)
3.0	90.21±3.27^a^	49.61±1.80^a^	572.09±10.11^a^
5.0	134.42±4.48^b^	73.93±2.47^b^	563.39±10.45^a^
7.0	126.06±2.84^b^	69.33±1.56^b^	535.17±20.63^a^
9.0	147.35±3.46^c^	81.04±1.90^c^	609.30±12.29^b^
11.0	161.87±2.88^d^	89.03±1.28^d^	642.88±9.91^c^
12.0	105.22±4.07^e^	56.61±2.58^e^	496.07±13.85^d^

Results were presented as mean ± standard deviation. Mean values with different letters were significantly different at p < 0.05.

#### Effect of temperature

The temperature had a significant effect on the specific surface area and AFB1 adsorption of MMS, as shown in Table 4, “temperature” referred to the reaction temperature and not the adsorption temperature. At temperatures < 80°C, the specific surface area of MMS was positively correlated with temperature. At 100°C, the specific surface area significantly decreased because the temperature affected the formation of micelles and the degree of silicate aggregation. An increase in temperature accelerated silicate polymerization, while hydrophobic groups on micelles become more stretched, contributing to the formation of an ordered mesoporous structure. However, excessively high temperatures could lead to an increase in the degree of silicate aggregation, a reduction in the MMS opening rate, and a decrease in the specific surface area [40]. Values for the unit adsorption capacity and AFB1 adsorption rate were greatest at 80°C. Therefore, 80°C was chosen as the optimum preparation temperature.

**Table 4:** Effect of reaction temperature on the specific surface area and adsorption to AFB1 of MMS.

Reaction temperature (°C)	Adsorption capacity (μg/g)	Adsorption rate (%)	Specific surface area(m^2^/g)
25	161.53±2.88^a^	88.84±1.80^a^	642.88±9.91^a^
40	154.35±2.44^b^	84.89±1.34^b^	660.74±4.98^a^
60	164.45±3.74^a^	90.45±2.06^a^	690.20±12.52^b^
80	171.98±3.60^c^	94.59±1.98^c^	730.98±12.81^c^
100	152.28±2.13^b^	83.75±1.17^b^	356.76±5.88^d^

Results were presented as mean ± standard deviation. Mean values with different letters were significantly different at p < 0.05.

### MMS properties

#### Magnetic separation characteristics

**Fig 1:**
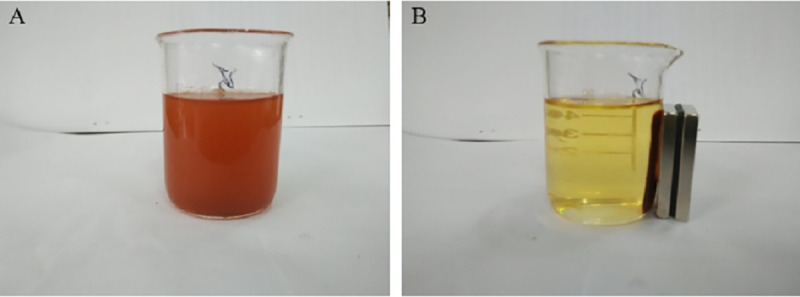
Magnetic separation of MMS (A: before adsorption; B: after adsorption).

Magnetic separation of MMS is depicted in Fig 1. A magnetic field could be applied to MMS to effectively separate it from the medium for recovery and recycling of the adsorbent, leaving the liquid medium depleted in the target contaminant.

#### Infrared spectroscopic analysis

**Fig 2:**
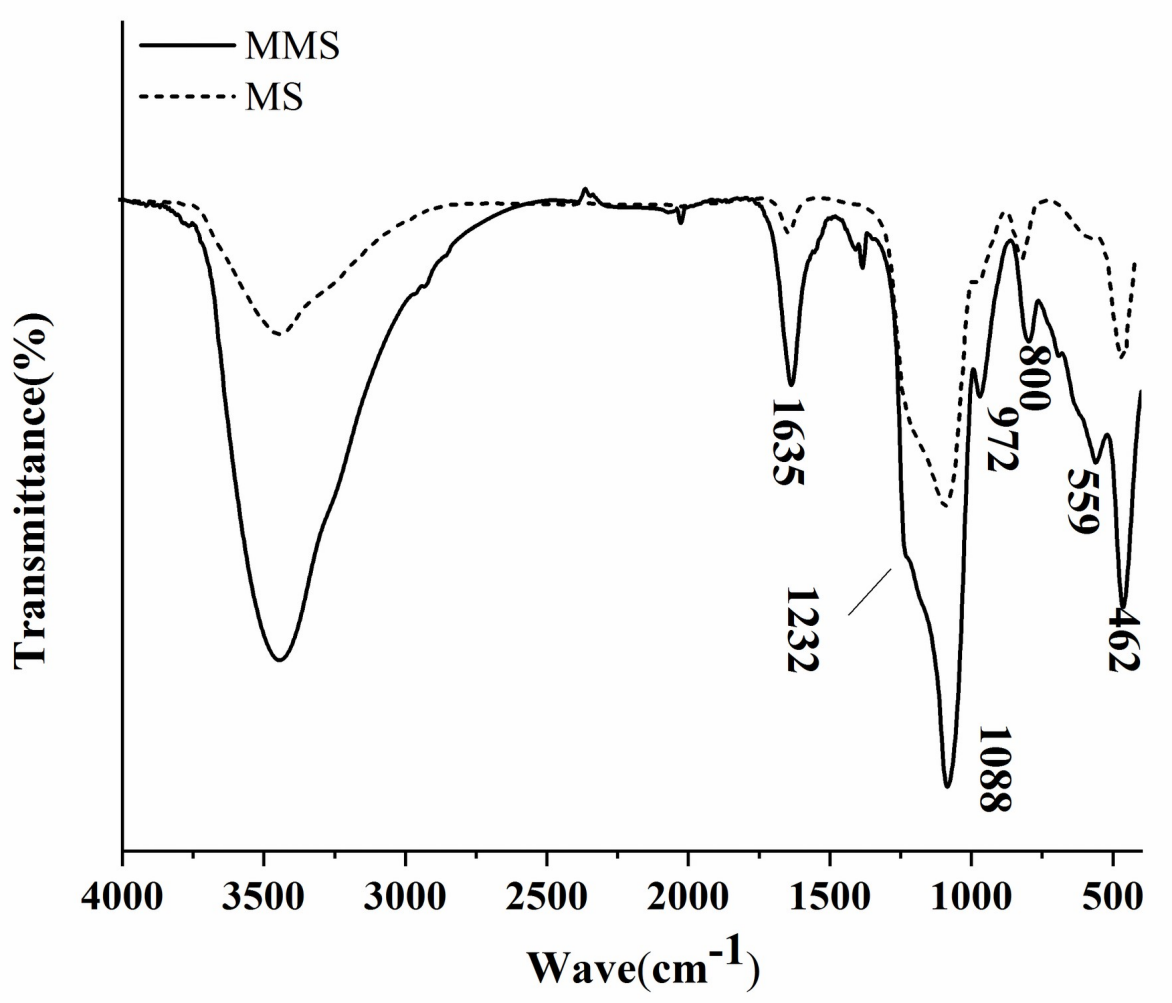
Infrared spectrum of MS and MMS.

The infrared spectrum of the sample is presented in Fig 2. The peaks at 1232, 1088, 800, and 462 cm^−1^ were assigned to Si–O stretching and bending vibrations. The peaks at 972 and 1635 cm^−1^ corresponded to bending vibrations for Si-OH groups [26]. The peak near 559 cm^−1^ could be attributed to Fe–O stretching vibration, indicating the presence of Fe_3_O_4_ [17], in agreement with the findings of Zahoor [41, 42] and the XRD results.

#### XRD analysis

**Fig 3:**
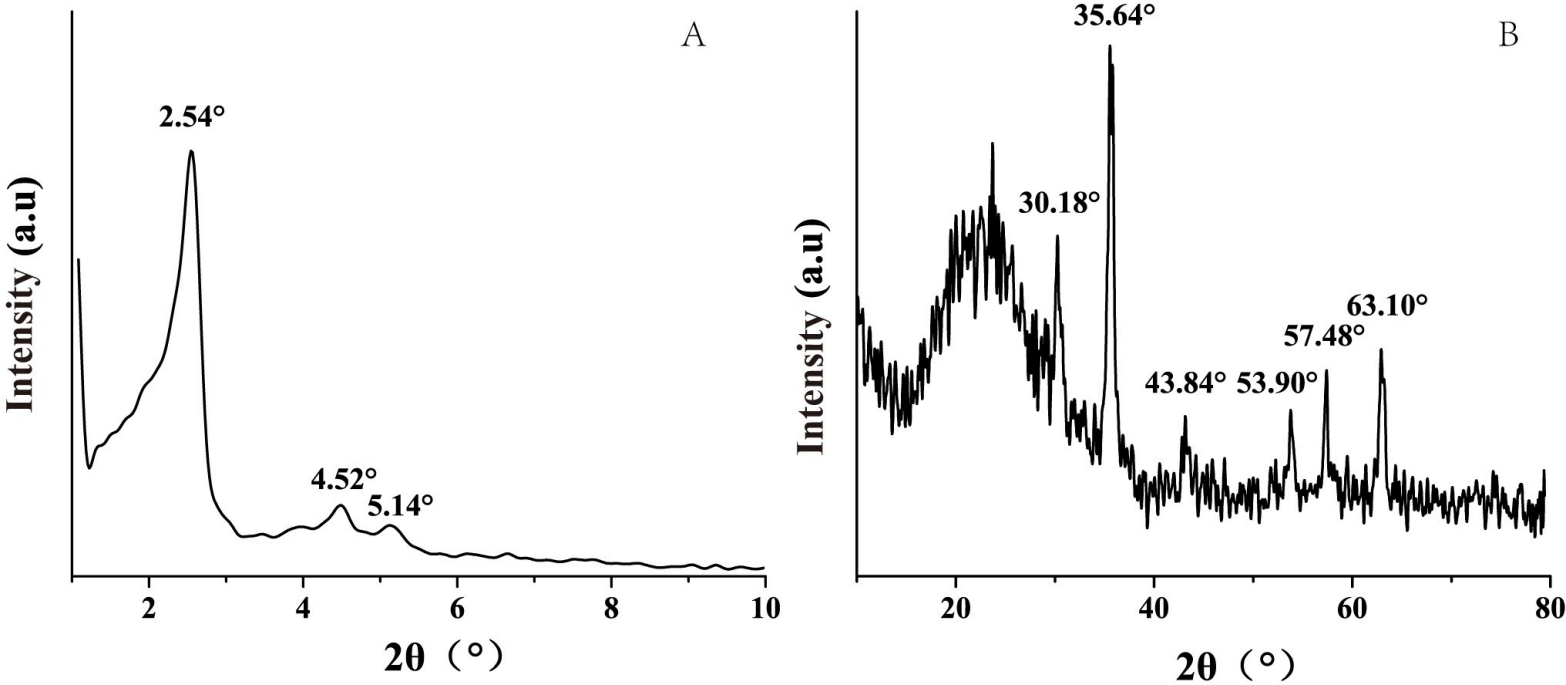
Small angle X-ray diffraction (A) and large angle X-ray diffraction (B) of MMS.

As observed in Fig 3, there were three obvious diffraction peaks at 2θ = 2.54°, 4.52°, and 5.14°, corresponding to the 100, 110, and 120 crystal planes, respectively. The diffraction peak for the 100 crystal plane was strong and narrow. We deduced that MMS had a highly ordered structure with six phases, as previously reported [43]. Fig 3 shows a broad diffuse peak at 2θ = 15°–30°, indicating an amorphous framework of SiO_2_ [44]. The diffraction peaks at 2θ = 30.18°, 35.64°, 43.84°, 53.90°, 57.48°, and 63.10° corresponded to the 220, 310, 400, 422, 511, and 440 crystal planes of cubic unit cells, respectively, indicating a magnetite structure as reported by Zahoor [41].

#### Static nitrogen adsorption-desorption analysis

**Fig 4:**
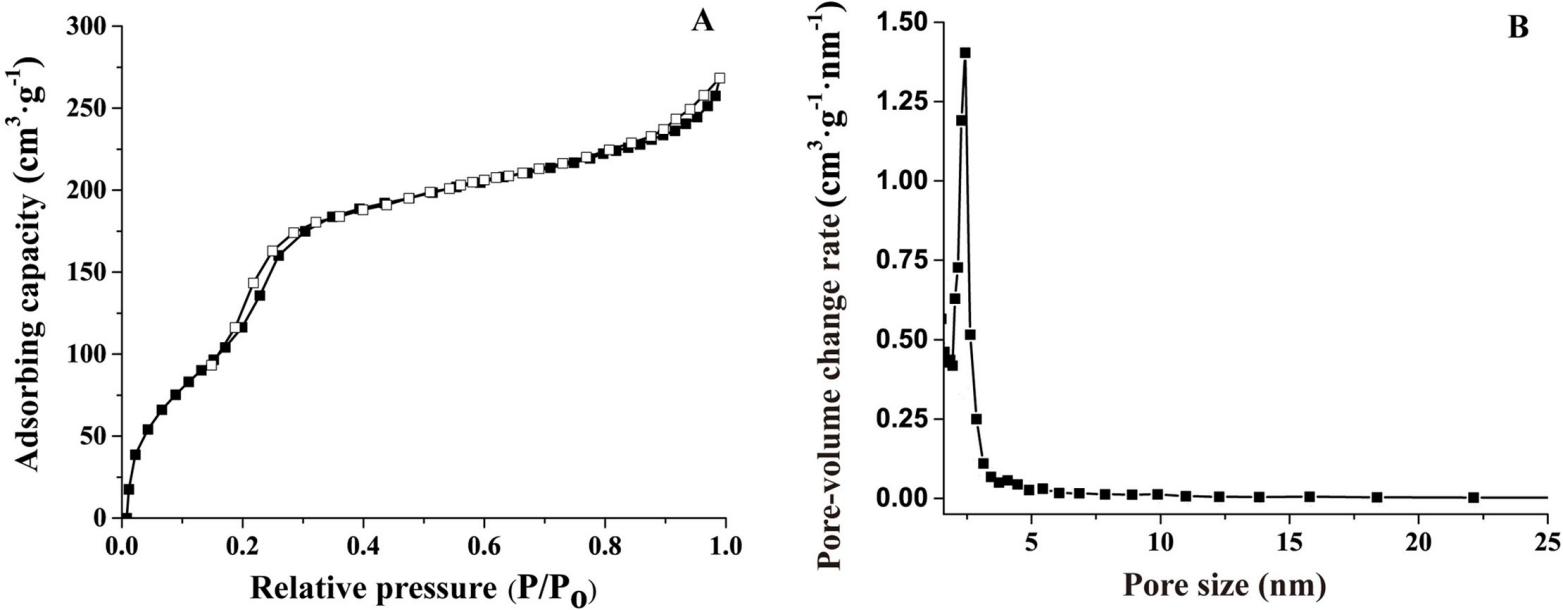
Nitrogen adsorption-desorption isotherms (A) and pore size distribution curve (B) of MMS.

Nitrogen adsorption-desorption isotherms and pore size distribution curves for MMS are presented in Fig 4. A type IV isotherm with a H1 hysteresis loop was observed, indicating that MMS was an MS material with a uniform pore structure. Fig 4 shows that MMS had a BET specific surface area of 730.98 m^2^/g and a pore diameter of 2.43 nm, which were consistent with the results from other studies showing that mesoporous materials had a pore size of 2–50 nm and high specific surface area (> 700 m^2^/g) [45, 46]. These included MMSN (859 m^2^/g) [18], MSN (872 m^2^/g) [47], MMS-PA (1089 m^2^/g) [48], and magnetite/silica/mesosilica microspheres (973.28 m^2^/g) [49].

### Optimization of AFB1 adsorption on MMS in oil

#### Effect of adsorption time

**Fig 5:**
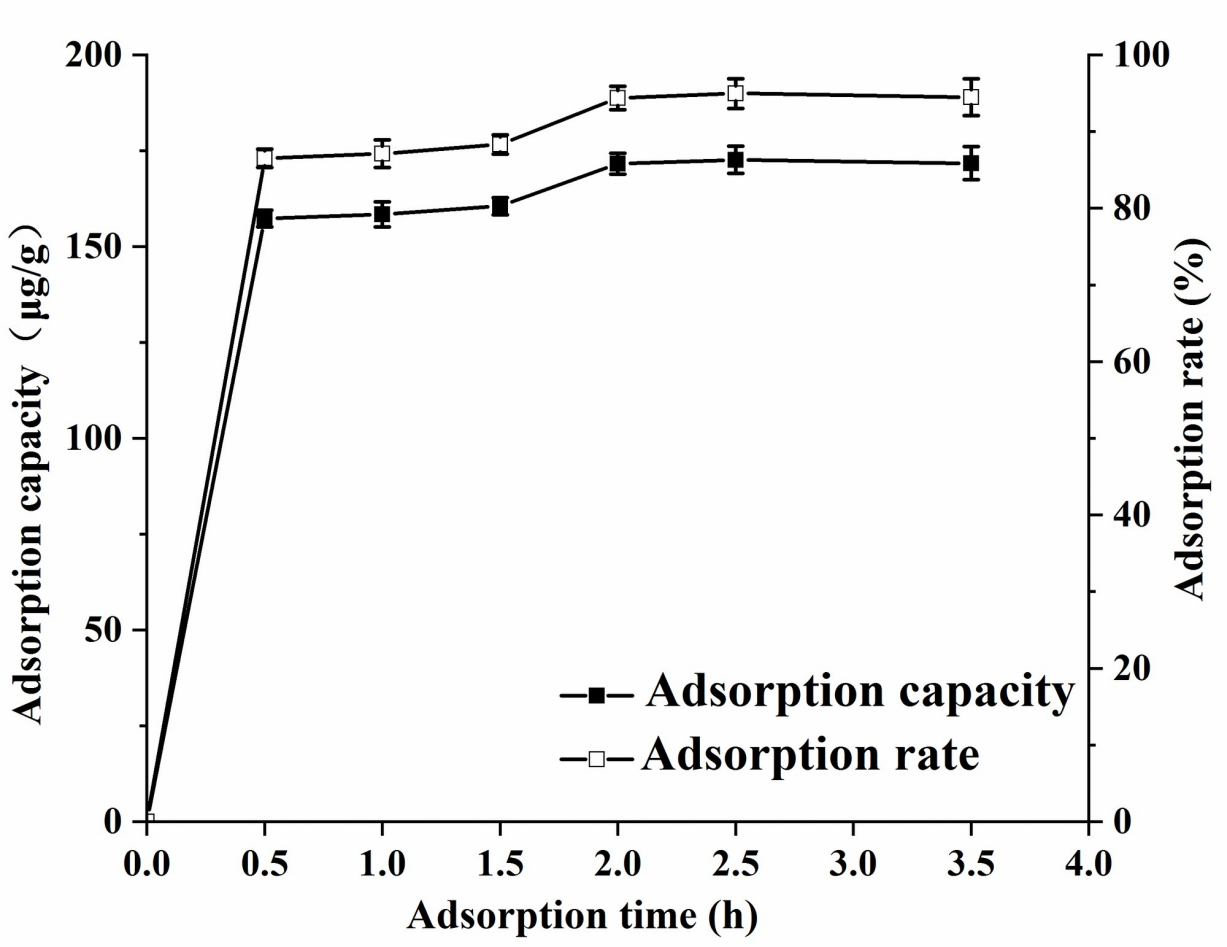
Effect of time on the adsorption of MMS to AFB1.

The AFB1 adsorption rate on MMS in oil system was investigated at 20°C. The medium was cooking oil containing 0.2 μg/mL AFB1. The unit adsorption capacity and AFB1 adsorption rate were determined after adsorption times of 0.5, 1.0, 1.5, 2.0, 2.5, and 3.5 h. Fig 5 shows that the AFB1 adsorption rate could be divided into two stages: rapid and slow adsorption. Rapid adsorption occurred from 0 to 0.5 h, during which the unit adsorption capacity and AFB1 adsorption rate rapidly increased with the adsorption time. After 0.5 h, the unit adsorption capacity and AFB1 adsorption rate increased slowly and reached a plateau at 2 h; the maximum unit adsorption capacity and AFB1 adsorption rate were 171.78 μg/g and 94.37%, respectively. The equilibrium time for AFB1 adsorption on MMS was similar to that for Novasil PLUS (calcium silicate) [42, 50], illustrating that our MMS had a high adsorption rate for AFB1.

The *R*^2^ value for the quasi-second-order kinetic model was 0.995, which was higher than for the quasi-first-order kinetic model (Table 5). The theoretical adsorption amount derived from fitting was close to the experimental value, indicating a better fit of the quasi-second-order kinetic model to AFB1 adsorption on MMS, and that the reaction involved might be chemisorption. According to Phillips [51], sodium calcium aluminum silicate had a strong adsorption capacity for AFB1 mainly because the aluminum could form a stable chelate with the electron-rich system (dicarbonyl group) of AFB1. Therefore, AFB1 adsorption on MMS might be a chemical adsorption process involving the formation of chelates between Fe^3+^ ions in MMS and the dicarbonyl groups of AFB1.

**Table 5:** The parameters of kinetic models.

Sample	q_e_ (experimental value) (mg/g)	Quasi-first-order dynamics			Quasi-second-order dynamics		
		k_1_ (min^-1^)	q_e_ (mg/g)	R^2^	k_2_ (g/mg•min)	q_e_ (mg/g)	R^2^
MMS	0.1720	1.0070	0.1654	0.9864	1.5328	0.1736	0.9953

#### Effect of temperature

**Fig 6:**
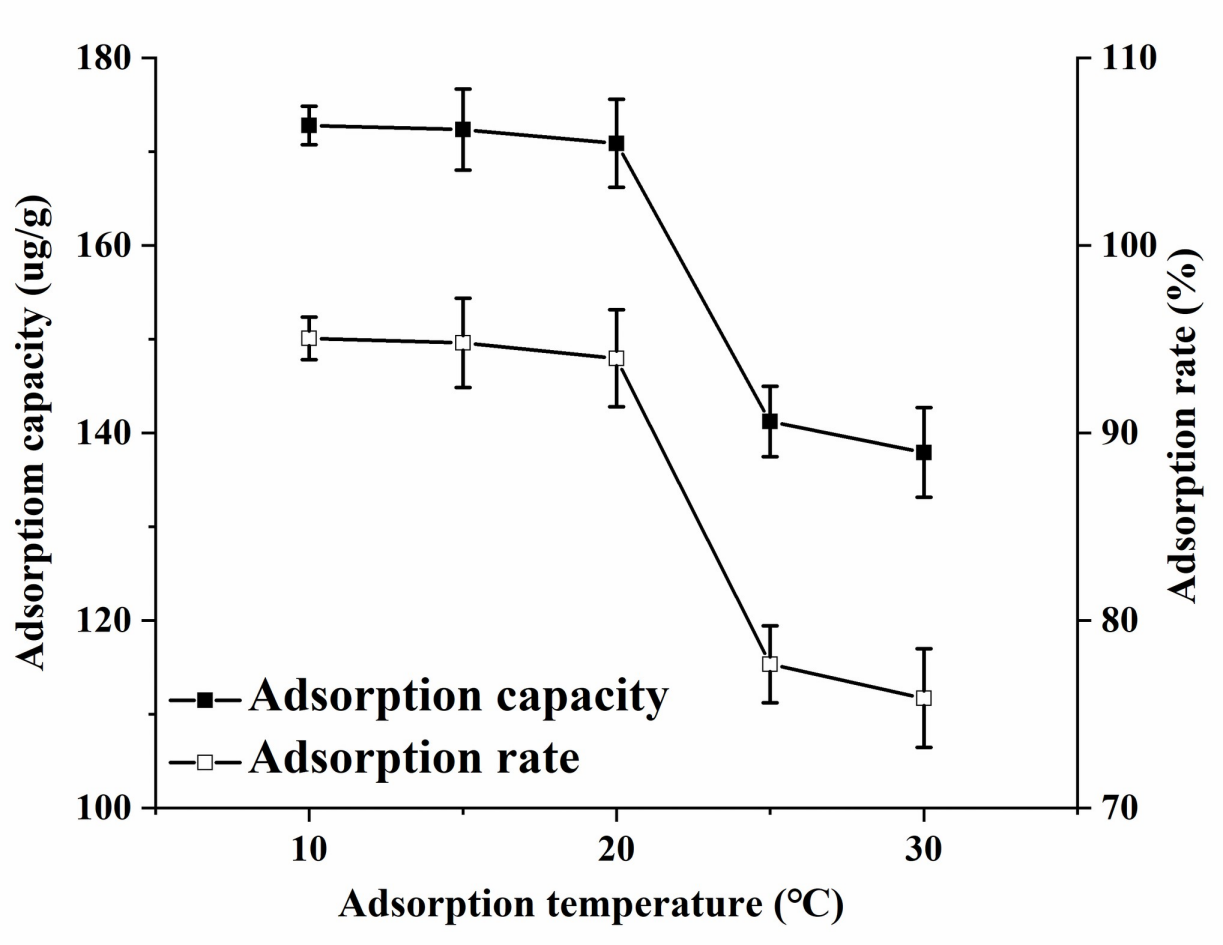
Effect of temperature on the adsorption of MMS to AFB1.

The AFB1 adsorption rate on MMS in oil was studied for a fixed adsorption time of 2 h. The medium was cooking oil containing 0.2 μg/mL AFB1. The unit adsorption capacity and adsorption rate at different temperatures were determined. Fig 6 shows that the unit adsorption capacity and AFB1 adsorption rate decreased with increasing adsorption temperature, indicating that AFB1 adsorption on MMS in oil was an exothermic reaction. At temperatures > 20°C, the unit adsorption capacity and adsorption rate decreased significantly with increasing temperature. Thus, the high temperature was not conducive to adsorption, which was consistent with the thermodynamics conclusion. For practical applications, it might be more appropriate to select 20°C as the adsorption temperature, which was close to room temperature.

### Isothermal AFB1 adsorption on MMS in oil

**Fig 7:**
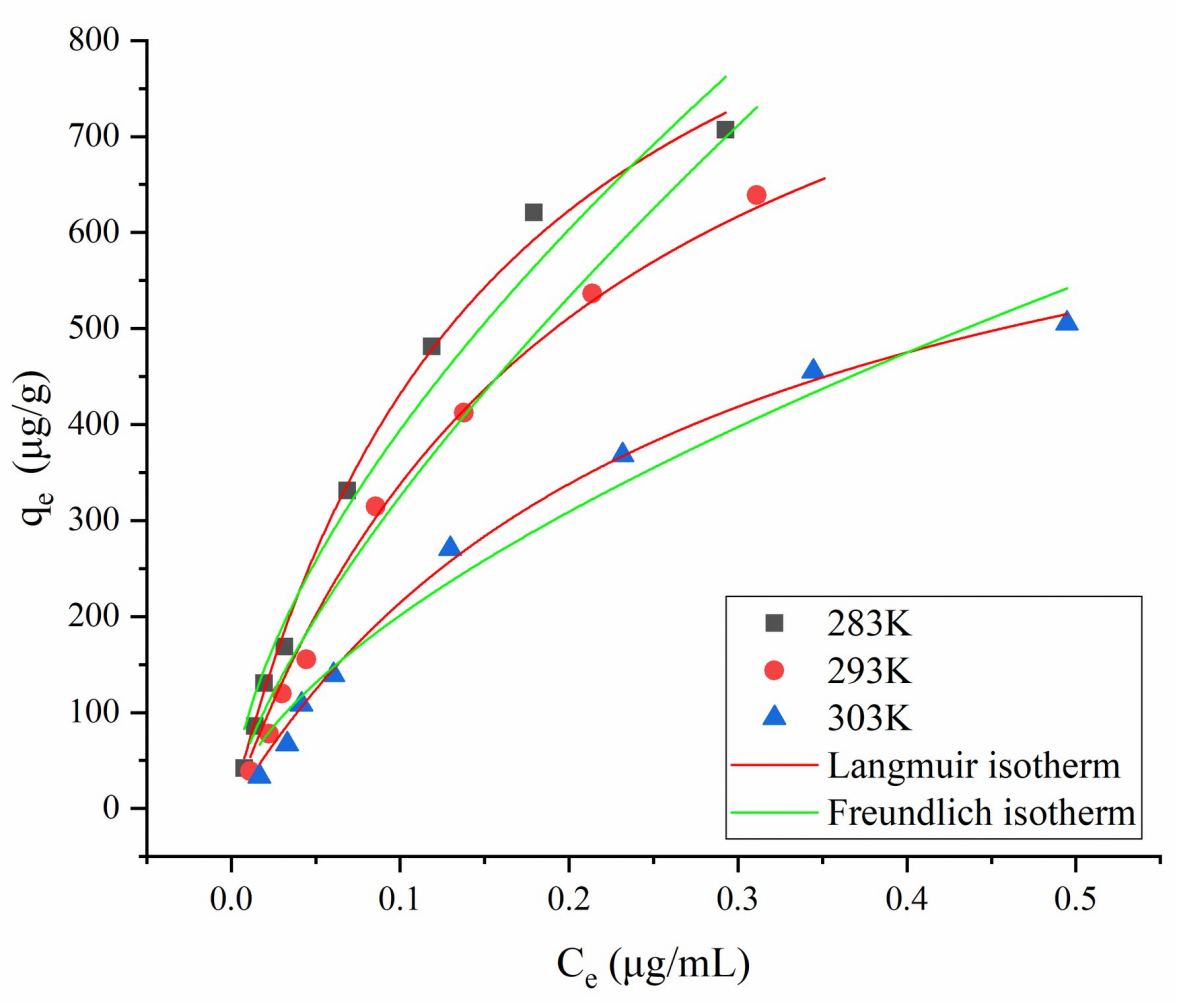
Isothermal AFB1 adsorption on MMS in oil.

Fig 7 shows isotherms for AFB1 adsorption on MMS in oil. The adsorption isotherms had an upward convex shape, confirming that AFB1 could easily adsorb on MMS. The isotherms were fitted to different isothermal adsorption equations and the regression parameters for Langmuir and Freundlich models were calculated [33]. Table 6 shows that *R*^2^ for fitting was greater for the Langmuir model than for the Freundlich model; all *R*^2^ values for the Langmuir model were > 0.98. These results demonstrated that AFB1 adsorption on MMS in oil conformed to the Langmuir model, with the assumption of monolayer AFB1 adsorption and uniform distribution of adsorption sites on the MMS surface. The maximum amount of AFB1 adsorbed on MMS according to the Langmuir isotherm was 1,118.69 μg/g at 10°C. The decrease of *K*_L_ with increasing temperature for the Langmuir model indicated that AFB1 adsorption on MMS was an exothermic reaction. For the Freundlich model, the values of 1/*n* at different temperatures were all < 1, indicating that AFB1 adsorption on MMS occurred easily. The values of *K*_F_ for the model were all large, indicating a high adsorption capacity of MMS for AFB1 and an abundance of adsorption sites on the MMS surface, which was similar to the findings of Hu et al. [17].

**Table 6:** Langmuir and Freundlich isotherm fitting equations of AFB1 in oil.

Temperature (K)	Langmuir isotherm fitting equations				Freundlich isotherm fitting equations			
	Equation	*q*_*max*_	*K*_*L*_	*R*^*2*^	Equation	*K*_*F*_	1/n	*R*^*2*^
283	*C*_*e*_*/q*_*e*_ = 0.0009*C*_*e*_+0.0001	1118.69	6.29	0.9958	ln(*q*_*e*_) = 0.61ln(*C*_*e*_)+7.39	1618.90	0.61	0.9694
293	*C*_*e*_*/q*_*e*_ = 0.0010*C*_*e*_+0.0002	1050.25	4.74	0.9963	ln(*q*_*e*_) = 0.71ln(*C*_*e*_)+7.43	1679.24	0.71	0.9755
303	*C*_*e*_*/q*_*e*_ = 0.0013*C*_*e*_+0.0003	798.58	3.67	0.9953	ln(*q*_*e*_) = 0.62ln(*C*_*e*_)+6.73	837.77	0.62	0.9685

### Calculation of the apparent thermodynamic parameters for AFB1 adsorption in oil

According to experiments, the isotherm for AFB1 adsorption on MMS in oil conformed to the Langmuir model; hence, *K*_a_ was obtained from *K*_L_ (Table 1) and the apparent thermodynamic parameters were obtained according to formulas (5)–(7). As shown in Table 7, the values of Δ*H* (−19.17 kJ/mol) and Δ*G* (−34.09, −34.61, and −35.15 kJ/mol at 283, 293, and 303 K, respectively) were negative. Δ*G* < 0 indicated that the AFB1 adsorption on MMS was a spontaneous process [20]. Δ*H* < 0 indicated that the AFB1 adsorption on MMS was an exothermic reaction [21] for which high temperature was not conducive [35], coinciding with the conclusion that temperature affected the AFB1 adsorption process. The absolute Δ*H* value of 19.17 kJ/mol was less than 40 kJ/mol, indicating that the reaction was dominated by physical adsorption [17]. Moreover, Δ*S* > 0 indicated that the increase in entropy might be mainly due to nondirectional AFB1 adsorption on the MMS surface.

**Table 7:** Apparent thermodynamic parameters of AFB1 adsorption in oil.

Temperature (K)	ΔG (kJ/mol)	ΔH (kJ/mol)	ΔS [J/(mol·K)]
283	-34.09	-19.17	52.73
293	-34.61		
303	-35.15		

In conclusion, the optimum adsorption conditions were 2h at 20°C, the MMS adsorption capacity was 171.98 μg/g and the adsorption rate was 94.59% with AFB1 initial concentration of 0.2 μg/mL. Compared with TEOS, rice husk ash was less expensive as a silica source, and MMS prepared from this raw material was easy to separate and it was reusable. This paper focused on MMS from rice husk ash for removal of AFB1 in oil, compared with other similar studies. Furthermore, this magnetic mesoporous material can be applied for removal of mycotoxins (AFB1 [41] and ochratoxin A [21]), toxic metals (Hg [19], Cd^2+^ [52], Cu, and Co [53]), dyes (methyl blue [25, 26]), pesticides (pyrethroid [54]), pharmaceuticals (minocycline [17]), and other emerging pollutants [40], and may be suitable for drug delivery [55] in the future.

## Conclusion

We investigated the preparation of MMS from rice husk ash and found that the optimum conditions were pH 11.0 and 80°C for 24 h. Under these conditions, the MMS specific surface area was 730.98 m^2^/g and the pore diameter was 2.43 nm. For AFB1 at an initial concentration of 0.2 μg/mL, the MMS adsorption capacity was 171.98 μg/g and the AFB1 adsorption rate was 94.59%. Magnetic particles were successfully incorporated in our MMS material, which allowed separation of the adsorbent from liquid material by applying a magnetic field, thus allowing the adsorbent to be recycled. The optimum adsorption conditions were 2 h at 20°C, the MMS adsorption capacity was 171.98 μg/g and the adsorption rate was 94.59%. The MMS adsorption isotherm fitted the Langmuir model. A quasi-second-order kinetic model showed a better fit to the MMS-AFB1 adsorption process and the thermodynamic parameters indicated that AFB1 adsorption on MMS was a spontaneous exothermic process with nondirectional adsorption. The study will serve as a guide for MMS practical applications.
